# Intracranial aneurysms and optic glioma – an unusual combination: a case report

**DOI:** 10.1186/s13256-016-0869-8

**Published:** 2016-03-30

**Authors:** Danilo De Paulis, Giancarlo Nicosia, Graziano Taddei, Hambra Di Vitantonio, Massimo Gallieni, Mattia Del Maestro, Soheila Raysi Dechordi, Renato Juan Galzio

**Affiliations:** 1 Department of Neurosurgery, San Salvatore City Hospital, L’Aquila, Italy; 2 Department of Life, Health & Environmental Sciences (MESVA), University of L’Aquila, L’Aquila, Italy

**Keywords:** Tumor, Aneurysm, Optic glioma

## Abstract

**Background:**

The coexistence of glial high grade tumors (glioblastoma, anaplastic astrocytoma) and cerebral aneurysms is common but the association with optic glioma is rare. The treatment of these associated lesions is problematic.

**Case presentation:**

A 36-year-old white woman presented to our institution with recurrent attacks of headache. Her preoperative radiological studies revealed a lesion of her left optic nerve with extension to the optic chiasma, an aneurysmatic dilatation of the left carotid bifurcation, a second aneurysm on the left of her middle cerebral artery, and a third one on her right anterior cerebral artery in the A2 tract. A left pterional approach was used to remove the tumor and clip the three aneurysmatic dilatations in a single stage.

**Conclusions:**

We report this unusual case of optic glioma associated to multiples aneurysms and review the pertinent literature. An adequate knowledge of this association is mandatory to plan the correct approach to avoid complications.

## Background

Optic pathway gliomas (OPGs) are predominantly classified as pilocytic astrocytomas: World Health Organization (WHO) Grade 1 [1]. Although 10 to 30 % are confined to unilateral optic nerve, the majority involves some combination of optic chiasm, optic tracts, hypothalamus and third ventricle. They can be present in any location along the course of the optic pathway, with significant visual morbidity [1]. In a minority of cases, focal neurological deficits can occur owing to tumor extension into adjacent areas of the brain.

The incidence of cerebral aneurysm with brain tumor has been documented (2.5 to 4.0 %) [2]. The coexistence of glial high grade tumor (glioblastoma, anaplastic astrocytoma) and cerebral aneurysms is common but the association with optic glioma is rare [3]. The treatment of these associated lesions is problematic; bleeding from the aneurysm after surgery for the tumor is considered the main cause of the poor outcome [2]. Despite this, the management of such cases has not been adequately addressed in the literature.

## Case presentation

A 36-year-old white woman presented to our institution with recurrent attacks of headache. She reported initial visual impairment in her left eye that was gradually worsening. A neurological examination showed left amaurosis, confirmed by a visual field test, associated to a severe optic atrophy at fundus oculi. No other deficits were disclosed and all routine laboratory findings were normal.

**Fig. 1:**
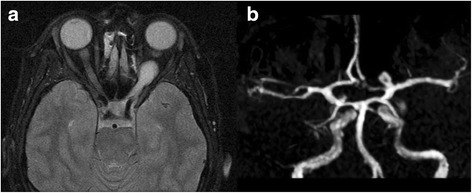
Preoperative magnetic resonance imaging showing, in T1-weighted sequence, the left optic glioma (**a**) and, in time-of-flight sequence, a suspect aneurysmatic dilatation (6×5 mm) on the left carotid bifurcation (**b**)

**Fig. 2:**
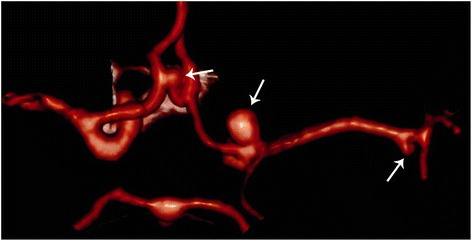
Preoperative computed tomography angiography three-dimensional reconstruction. The reconstruction shows the three aneurysms (*arrows*); it confirms the aneurysm of the left carotid bifurcation and demonstrates the second aneurysm on the left of the middle cerebral artery and the third one on the right anterior cerebral artery in the A2 tract. Notice that two aneurysms were not visible in time-of-flight-magnetic resonance imaging reconstruction in Fig. 1

A magnetic resonance imaging (MRI) study of her brain revealed a lesion of her left optic nerve (22×13 mm) with extension to the optic chiasma and a suspect aneurysmatic dilatation (6×5 mm) of the left carotid bifurcation (Fig. 1). A computed tomography (CT) scan of her brain was performed with angiography and a three-dimensional reconstruction confirmed an aneurysmatic dilatation of the left carotid bifurcation, and it revealed a second aneurysm on the left of her middle cerebral artery and a third one on her right anterior cerebral artery in the A2 tract (Fig. 2).

A left pterional approach was used to remove the tumor and clip the three aneurysmatic dilatations in a single stage. The tumor was removed by cutting proximally and distally the optic nerve that could not be dissected from the lesion. Histopathological examination confirmed the diagnosis of astrocytoma grade I.

**Fig. 3:**
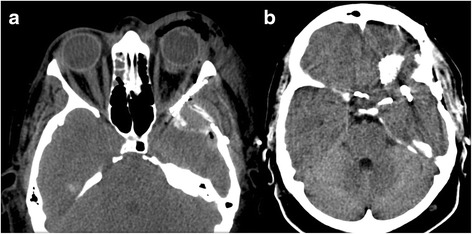
Postoperative computed tomography scans after removal of the tumor and clipping of the three aneurysmatic dilatations in a single stage (**a**, **b**)

Her postoperative course was satisfactory without complications (Fig. 3), except for the left amaurosis which was present preoperatively.

## Discussion

Cerebral aneurysm has been associated with a wide variety of tumor types, including meningioma (29.3 to 44 %), glioma (27.5 to 38 %), pituitary adenoma (11 to 20.6 %), and with a lesser frequency lymphoma, craniopharyngioma, chordoma, epidermoid tumor, dermoid tumor, and choroid plexus adenoma [2–5]. Initial symptoms were caused by the tumor (54 to 78 %) or by the rupture of the aneurysm (17 to 45 %). Symptoms from both were recorded in 6 % of cases [2, 3].

The association of brain tumors with cerebral aneurysms raises the question of whether a causal relationship exists. Several hypotheses have been proposed to explain the possible association.

An increase in the directional blood flow due to a higher blood supply to a meningioma or malignant glioma may induce secondary changes in the arterial wall and thus facilitate the formation of aneurysms [2]. The role of growth hormone in promoting vascular disease can be a predisposing factor [2]. However, some authors state that hormones have no effect on the formation of aneurysms [6].

In aneurysms that are within or adjacent to a brain tumor, histopathological examination for infiltration of the aneurysm wall from neoplastic cells has been demonstrated [2, 5]. Development of aneurysms by tumor invasion is linked to the histology of the tumor [3]. Vessel wall invasion by tumor cells may occur in glial tumors, lymphomas, and pituitary tumors even if a rapid growth of the tumor does not give enough time to produce mechanisms for the formation of an aneurysm [2, 3]. Vessel wall invasion by tumor cells is not reported in meningioma and in low grade gliomas [6]. However, in these cases genetic alterations may underlie the development of multiple aneurysms [7]. In the literature, it is shown that the messenger ribonucleic acid expression of tumor necrosis factor-α (TNF-α) and Fas-associated death domain protein (FADD) are increased in human cerebral aneurysms, suggesting a key role for these cytokines in promoting inflammation and subsequent apoptosis of cerebral vascular cells with development of an aneurysm sac [8]. Furthermore, immunostaining studies on vascular endothelial growth factor (VEGF) suggest that the VEGF gene plays a role in the pathogenesis and enlargement of cerebral aneurysms [9].

In postoperative treatments for tumors, the destructive effects of irradiation on blood vessels are well known but rare; only 21 cases have been reported in the literature [10]. Radiation-induced vascular changes develop as early as 4 months or as late as 23 years after radiotherapy [11, 12].

Intratumoral, subdural or subarachnoid hemorrhage (SAH) may occur in primary brain tumors. Spontaneous SAH is not common in malignant cerebral tumors, but the presence of aneurysms must therefore be investigated if it occurs [6]. The prognosis is linked mainly to the nature of the tumor with unruptured aneurysms, and to the evolution of SAH in patients with ruptured aneurysms [3]. Death was mainly due to progressive evolution of the malignant tumor (46 %) or fatal aneurysmal bleeding (23 %). The localization and the pathological nature of the tumor are the most important prognostic factors [13]. Association of both lesions does not worsen the outcome [3].

If the aneurysm is unruptured, the treatment options include conservative management, surgical clipping and tumor excision either simultaneously or sequentially, or endovascular coiling followed by tumor removal [2, 13]. Clipping for ruptured cerebral aneurysm and resection of the brain tumor is the best treatment option. The lesion producing the presenting symptoms should be treated first when a single approach to both is unfeasible [14]. Decisions regarding exclusion of an incidental aneurysm should be balanced against the risks of the procedure [3]. The incidental aneurysm may be managed conservatively in patients with coincidental tumor and aneurysm if it is not in the field of surgery for the tumor [2].

The presence of an incidental aneurysm close to or embedded within a tumor makes the surgical excision of the tumor hazardous. The lesional resection can be performed following endovascular coil embolization of the aneurysms to reduce the risk of intraoperative rupture and to enable total tumor removal [13]. In cases in which the tumor has an unfavorable prognosis, the best optional treatment for the aneurysm is not surgery to protect the patient from its complications.

The rate of aneurysm rupture during the intraoperative period must be dealt with by taking precautions and even more so when there is a comorbidity, such as a neoplasia, which can increase difficulties, extend, and potentially complicate the procedure. In these cases, the perioperative anesthetic management can have a significant effect on global neurological results by monitoring many factors, including the induction of anesthesia, the presence or absence of burst suppression, use of volatile agents/muscle relaxants, hemodynamic parameters, and temperature. In any case, these parameters must be assisted by intraoperative neurophysiological monitoring with EEG and evoked potentials [15].

## Conclusions

In the literature, all cases of cerebral aneurysm associated with optic glioma referred to patients with a history of treatment with radiotherapy [12], while in this case the unruptured aneurysms were an incidental finding. The prevalence of incidentally discovered brain tumor and cerebral aneurysm has been increased by an improvement in high-resolution imaging techniques. So an adequate knowledge of this association is mandatory to plan the correct approach to avoid complications.

## Consent

Written informed consent was obtained from the patient for publication of this case report and any accompanying images. A copy of the written consent is available for review by the Editor-in-Chief of this journal.
